# Neurodevelopment and climate change

**DOI:** 10.1016/j.jped.2024.10.005

**Published:** 2024-11-22

**Authors:** Magda Lahorgue Nunes, Antônio José Ledo Alves da Cunha

**Affiliations:** a Pontifícia Universidade Católica do Rio Grande do Sul (PUCRS), Escola de Medicina, Porto Alegre, RS, Brazil; b Instituto do Cérebro (InsCer), Porto Alegre, RS, Brazil; c Universidade Federal do Rio de Janeiro (UFRJ), Faculdade de Medicina, Departamento de Pediatria, Rio de Janeiro, RJ, Brazil

**Keywords:** Childhood, Adolescence, Environment, Climate change, Neurodevelopment

## Abstract

**Objective:**

This article aims to assess the impact of climate change, a reality already present on the neurodevelopment of both neurotypical and atypical children.

**Data sources:**

A narrative review of the literature was carried out based on articles available in the PubMed database, published in the last five years using the keywords neurodevelopment and climate change, as well as websites of organizations dedicated to childhood such as UNICEF, the American Academy of Pediatrics and the Center for Developing Childhood at Harvard University.

**Summary of findings:**

Children and adolescents are more directly affected by the effects of climate change due to their developmental stage and greater vulnerability. Prolonged exposure to air pollutants can affect brain development, resulting in cognitive and behavioral problems. Extreme weather events, such as floods, cyclones, and heat waves, can destroy essential infrastructure such as schools and hospitals, interrupting the educational process and access to health care. Changes in rainfall patterns and extreme droughts can affect food production, leading to malnutrition and food insecurity. Direct experience of natural disasters can cause stress and psychological trauma, affecting children's emotional and mental well-being.

**Conclusions:**

Studies clearly demonstrate the potential impact of climate change on the neurodevelopment and mental health of children and adolescents. This topic should be part of the current agenda of pediatricians, not only treating the resulting illnesses but mainly acting on the front line and supporting proposals to attenuate the environmental disaster that has already occurred.

## Introduction

Living with the consequences of climate change will certainly be one of the greatest challenges for future generations. It is widely known that in recent centuries, the environment has been affected by human actions. Deforestation, environmental pollution, fires, and inadequate disposal of toxic waste are responsible for the climate catastrophes that have been occurring with alarming frequency in all regions of the planet. Science has an important role not only in proving these phenomena and alerting the population, but also in trying to attenuate or propose adaptations that can slow down or reduce these effects.^1^, ^2^, ^3^

Climate change can have an impact on several aspects of the population's health, especially children during their development. The impact of extreme weather events (heat waves, blizzards, storms, floods), pollution, and rising sea levels impact individual behaviors such as diet, physical activity, sleep, substance abuse, and lack of preventive health care.^1^

The Health Metrics and Evaluation Institute, which conducts studies on the Global Burden of Diseases, includes environmental risk factors such as air pollution, lead exposure and climate change in its measurements. They believe there is a lack of studies in regions with high exposure to pollution, and that more effective methods for detecting lead poisoning are needed. In future analyses and publications, top priority should be given to incorporating the role of neurotoxic substances, endocrine-metabolic disruptors (hormone-related cancer, infertility, reproductive dysfunction, congenital malformations, obesity, diabetes and neurobehavioral disorders) and climate change so that preventive measures can be established in regions at higher risk.^4^

The American Academy of Pediatrics calls on its members to advocate for solutions to climate change, as they believe that children's physical and mental health is seriously threatened by extreme temperatures (heat waves and fires), disruption of ecological cycles and, consequently, of the communities. It warns this impact is greater on children from socially disadvantaged groups, increasing inequality.^5^^,^^6^

Several global organizations and international agencies, such as UNICEF, have been taking action on this issue as a priority. UNICEF, which advocates that the climate crisis is a Child Rights crisis, recently presented the Children's Climate Risk Index.^7^ This is the first comprehensive analysis of climate risk from a child's perspective. The Risk Index classifies countries based on the children's exposure to climate and environmental shocks, such as cyclones and heat waves, as well as their vulnerability to these shocks, based on access to essential services. The Index shows that approximately one billion children and adolescents – almost half of the world's 2.2 billion girls and boys – live in one of the 33 countries classified as being at extremely high risk. The Index further reveals that 240 million children and adolescents are highly exposed to coastal flooding, 330 million to riverside flooding, 400 million to cyclones, 600 million to vector-borne diseases, 815 million to lead pollution, 820 million to heat waves, 920 million to water scarcity, and one billion to extremely high levels of air pollution.^7^ These children and adolescents face a deadly combination of exposure to multiple climate and environmental shocks with high vulnerability due to inadequate essential services, such as water and sanitation, health and education.

Although the World Health Organization (WHO) has already defined parameters for adequate air quality, it can be observed that almost the majority of the global population lives under continuous exposure to concentrations of pollutants, such as nitrogen dioxide, lead, carbon monoxide, ultrafine particles, and sulfur dioxide, at levels higher than acceptable ones.^8^

In turn, Neurodevelopment is defined as the set of skills through which the child interact with their surroundings, from a dynamic perspective, according to their age, their degree of maturity, their intrinsic biological factors, and stimuli from the environment.^3^

Neuropsychomotor development (NPMD) is a continuous and orderly process of differentiation, defined by behavioral patterns that accompany the child from birth. NPMD is assessed through the evolution of these behavioral patterns, which are related to chronological age (or corrected age in the case of preterm infants) and that are a defined response of the central nervous system (CNS) to a specific situation. The evolution of these patterns is related to CNS integrity and maturity. The acquisition of cognitive functions such as attention, memory, executive functions, and language, which will be perfected in adolescence, begins to be organized during the first years of life.^9^^,^^10^

Based on the innovative scientific knowledge that identified the period between gestation and the second year of life as an important window for interventions with a long-term impact on children's health and development, a global campaign was launched, based on a North American initiative, aimed at drawing attention to the “First 1000 Days of Life” (gestation 270 days + 1–12 months 365 days + 1–2 years 365 days), endorsed by UNICEF and medical societies and non-governmental organizations focused on the care of children in several countries.^9^^,^^11^

The climate crisis can have significant impacts on child neurodevelopment. Children and adolescents are particularly more sensitive to the effects of climate change due to their stage of development and greater physical vulnerability. Some of the main impacts include **air pollution**. Prolonged exposure to air pollutants can affect brain development, resulting in cognitive and behavioral problems.^12^ Moreover, **extreme weather events** such as floods, cyclones, and heat waves can destroy essential infrastructure such as schools and hospitals, disrupting the educational process (with direct consequences for learning) and access to health care.^12^

Another aspect to be taken into account is the risk of **food insecurity**, leading to malnutrition, which can occur as a consequence of reduced food production/supply, resulting from both changes in rainfall patterns and extreme droughts.^12^ Regarding **stress and trauma**, exposure to natural disasters can cause psychological stress and trauma, affecting children's emotional and mental well-being.^12^ These factors, when combined, can impair children's cognitive, emotional, and physical development, highlighting the urgent need for policies and actions aimed at protecting young people from the impacts of climate change.^13^

Climate change has been progressively occurring in recent years and the authors have been experiencing, with increasing frequency and intensity, extreme heat waves, floods and large-scale fires. These events directly affect large portions of the global population and their impact on the first 1000 days of children's lives is still a topic that needs to be further investigated.

This article aims to assess the impact of climate change, a reality already present in the environment, on children's neurodevelopment.

## Impact of prenatal exposure

During pregnancy, adverse environmental issues can have a major impact on the fetus and the newborn, as well as in the longer term, during development. Examples include the disaster with the mercury deposition in Minamata Bay in Japan in the 1950s and 1960s, leading to serious neurological conditions (ataxic encephalopathy) and fetal microcephaly; and accidents involving exposure of the population to high doses of radiation, such as the Chernobyl nuclear accident in Russia in 1986 and the Cesium-137 accident in Goiânia in 1987; in these cases, a higher risk of miscarriage and fetal microcephaly was observed.

More recently, several fires have occurred in different parts of the world due to prolonged droughts and illegal burning. Murphy et al., in a systematic review study, raised the question of the harmful effects of exposure to smoke from forest fires during pregnancy and its association with low birth weight and increased risk of preterm birth. Prenatal stress resulting from catastrophic events is also likely to be associated with adverse perinatal outcomes, such as prematurity and low birth weight. Changes in DNA methylation are potential epigenetic mechanisms that could explain the association between smoke particle inhalation and prenatal stress and the consequent impact on the development of respiratory diseases (asthma and upper respiratory tract infections) throughout childhood.^14^

A systematic review study assessed the impact of typical summer month temperatures during pregnancy on the fetus and newborn, in addition to mental health outcomes (schizophrenia and anorexia nervosa). A correlation was observed between higher temperatures in a critical period of pregnancy (between the 3rd and 8th weeks) and a higher risk of congenital malformations. Possible explanations would be the increase in intrauterine temperature-altering enzymatic activity, cell proliferation and neuronal migration. In this same study, the results regarding the two mental health outcomes evaluated were controversial.^15^

More recent studies provide evidence that exposure of pregnant women to environments with polluted air can affect the circuits responsible for the fetus’ hypothalamic development, causing metabolic changes that will be observed in childhood and throughout life. The pathophysiological mechanisms are not yet fully understood, but it is likely that pollution triggers a systemic inflammatory process with an increase in circulating cytokines, which subsequently enter the brain circulation, causing neuroinflammation and neurotoxicity.^16^

Studies in humans have shown that the activation of oxidative stress and inflammatory pathways during pregnancy can alter the blood-placental barrier, allowing toxic substances such as iron, copper, lead, and black carbon to reach fetal tissues.^8^

## Impact on neurodevelopment

After birth, the pathways through which air pollution can reach the central nervous system are respiratory (inhaled particles enter the lungs, pass through the alveoli, enter the bloodstream and eventually penetrate the brain) and nasal (inhaled particles are directly transported to the brain via the olfactory nerve and bulb).^8^

Air pollution can affect neurodevelopment through two main mechanisms. The first is based on exposure to nitrogen dioxide and suspended particles with a diameter of <2.5 micrometers (PM 2.5), where these substances lead to neuronal damage and loss in structures of the prefrontal cortex, olfactory bulb and midbrain through an inflammatory process. This mechanism would be associated in the medium term with attention deficit disorder (ADHD) and later with Alzheimer's disease. The second would occur through the alteration of the microbiota. Components of the intestinal microbiota, such as *Bifidobacterium infantis*, regulate central neurotransmitters, such as serotonin, and can modify its precursor, tryptophan. Short-chain fatty acids are also produced by the microbiota and could affect neurodevelopment, as well as inflammatory signaling agents, such as cytokines. Studies suggest that depression, anxiety, autism and ADHD may be associated with microbiota dysbiosis.^17^^,^^18^

Ming & Ray discuss the impact of ecosystem disruption and its impact on health and neurodevelopment. With climate change impacting the environment, the authors are frequently exposed to new microorganisms that direct the immune system to defend itself through an inflammatory reaction with the creation of new antibodies. This process, which is initially protective, when occurring chronically could be related to the increase in “allergic reactions” and encephalitis of unknown etiology. Continuous exposure to antibiotics and disinfectants can result in changes in the microbiota of the skin, oropharynx, intestine and other organs. The exclusion of symbiotic bacteria can be disruptive to this microenvironment, resulting in digestive pathologies and also impacting the development of the central nervous system and behavior regulation.^19^, ^20^, ^21^

It is important to emphasize that knowledge about the impact of air pollution on peripheral inflammatory and neurotoxic mechanisms is almost entirely based on the results of experiments carried out in animal models. These studies highlight the following potential mechanisms: changes in glial cells facilitating neuronal damage; production of inflammatory cytokines by microglia and astrocytes leading to myelination changes and neurodegeneration; and increased permeability of the blood-brain barrier.^8^

The evidence in humans is based on structural neuroimaging studies (brain magnetic resonance imaging) that show an association between air pollution and cortical thickness (gray matter volume) of the frontal, parietal, temporal and basal ganglia regions. These structural changes can impact several neurodevelopmental functions, such as executive functions, motor system, and behavioral changes.^8^

## Impact on cognition

More recent studies also speculate the impact of air pollution on cognitive processes. The findings suggest a direct association between pollution and reduced volume of brain structures in addition to the weakening of connectivity pathways, resulting in an impact on cognitive functions (reasoning, problem-solving, visuospatial organization).^22^ This relationship between air pollution and cognitive decline seems to be closely related to the type of pollutant, the pollutant level and the time of exposure.^23^

## Impact on mental health

The climate crisis also has an impact on the mental health of children and adolescents. Some characteristics observed are increased feelings of sadness, changes in appetite and sleep, difficulty concentrating and a feeling of disconnection from the environment. The pathophysiological mechanisms are not yet fully understood; however, young people have varied perceptions about climate change and these perceptions are directly related to their social context. Feelings of immense concern have been observed more frequently and have been renamed with the term eco-anxiety. Today's children and young people are already experiencing these transformations, both through direct exposure and through information disseminated by the media. Being aware that these situations can intensify and negatively impact the quality of life on the planet is certainly frightening and corroborates these symptoms.^24^

A systematic review study, including mostly adults, reports a direct association between increased pollution levels and internalizing symptoms (depression and anxiety), as well as structural and functional changes (oxidative stress, changes in neurotransmitters and neuromodulators) in brain regions such as the prefrontal cortex, amygdala and hippocampus, which could explain these findings.^25^

Children are undoubtedly more susceptible to climate change due to the amount of time they are exposed to nature, playing in parks and squares. Gislasonet et al. developed a systematic review study with the aim of elucidating three main questions: a) What is the direct and indirect impact of climate change on mental health?; b) What are children's and adolescents' perceptions of climate change? and c) Would taking part in mitigation actions increase resilience? Regarding the first question, they concluded that climate change brings extreme challenges to children, as it affects their right to life, health, food availability, water quality, and home safety (many families have to move to less inhospitable regions). Another direct aspect is the impact of climate change on increasing inequality. Regarding the other objectives, they observed that inclusion and participation in community actions to mitigate climate change results in greater resilience of those involved. In addition to providing a greater connection with the environment (land, water, animals), it also has positive effects.^24^

Silveira et al. studied the impact of fires on the mental health of a population sample of 725 California residents who were exposed in different ways to this environmental disaster. The assessment was carried out using validated scales for post-traumatic stress, anxiety, depression, and resilience, administered six months after the event. Although the recruited population was at the pediatric age limit (the youngest ones were around 18 years old), the results show that direct exposure to fires significantly increased the risk of mental illnesses, especially depression and post-traumatic stress. A history of trauma during childhood and sleep disorders were predictors of a worse prognosis in terms of mental health. Resilience (self-reported) and the practice of mindfulness were protective factors, reducing levels of anxiety and depression.^26^

A longitudinal study conducted with 145 adolescents (9–13 years old) analyzed depression scores, measured at three different times, and the pollution load in the place of residence. They observed a deficit in the regulation/modulation of emotions and an increase in depressive symptoms in association with living in communities with a higher pollution load.^27^

## Impact on neurological diseases (epilepsy)

Climate change can influence the frequency of seizures in people with epilepsy through the induction of precipitating factors. Among these are fever, stress, and sleep deprivation (which can occur as a consequence of extreme temperatures, both excessive heat and cold). Infectious diseases transmitted by vectors (e.g.: Dengue fever, Zika, Chikungunya, Malaria) or parasitic diseases (such as neurocysticercosis) can increase their incidence in very hot temperatures. All of these tropical diseases have epileptic seizures as their main neurological clinical expression. Although the relationship between epilepsy and climate change is complex, multifactorial and indirect, experimental studies carried out in animal models show the seasonality of seizures, as well as the impact of increased body temperature reducing the threshold for seizures and increasing the risk of brain damage. Several mechanisms could explain the greater susceptibility to seizures with increased body temperature, among which are genetic susceptibility, ion channel permeability changes, pro-inflammatory immune system activation (interleukin 1b and tumor necrosis factor) and the induction of hyperventilation resulting in alkalosis. Climate change could also affect the action of anti-seizure drugs, either due to storage issues (excessive humidity or heat or exposure to sunlight) or due to excessive sweating leading to reduced plasma concentrations of these drugs.^28^

It is also important to emphasize that some epileptic syndromes are particularly affected by climate change, in which case the susceptibility to seizures increases with fever and higher temperatures. Examples include Dravet syndrome (related to the SCN1A sodium channel mutation) and other epileptic encephalopathies such as SCN2A (neonatal form), ARX (Xp22.13), CDKL5 (Xp22), SL25A22 (11p15.5), and STXBP1 (9q34.1).^29^^,^^30^

Given the relevance of the topic, the “International League Against Epilepsy” (ILAE) has created a specific committee to monitor this issue and conduct studies on this topic.^30^

## Final considerations

Children are much more exposed than adults to the potentially harmful effects of climate change. This is due to aspects specific to a developing organism (physiology), as well as habits of greater exposure to external environments (outdoors), and the distribution of food and water per kilogram of weight. Several studies suggest that different environmental aspects resulting from climate change can impact children, including air pollution, excessive heat, floods and hurricanes and the resulting food, nutritional and housing insecurity that may result from these events. Moreover, exposure to new infectious agents and the direct and indirect impact on mental health are relevant aspects.

Climate change is no longer an agenda to be considered by future generations, as it is already affecting several ecosystems and impacting all our lives. Although the human body has the ability to adapt to environmental issues, this is a long-term process.

It is the pediatrician's role to advocate for solutions to the climate crisis, encouraging the use of renewable energy, planting trees and creating green spaces, promoting access to healthy foods, encouraging the use of public transportation and the construction of safe cycleways, as well as supporting the construction of affordable and energy-efficient homes.

Immediate global action is essential, through effective awareness campaigns among the population and greater regulation by government environmental protection agencies, to at least try to curb these extreme changes. Several medical and other scientific entities are already taking a stand, by both issuing warnings and proposing preventive measures.

## Conflicts of interest

The authors declare no conflicts of interest.
